# Ensemble Machine Learning Approaches for Bathymetry Estimation in Multi-Spectral Images

**DOI:** 10.3390/geomatics5030034

**Published:** 2025-07-22

**Authors:** Kazi Aminul Islam, Omar Abul-Hassan, Hongfang Zhang, Victoria Hill, Blake Schaeffer, Richard Zimmerman, Jiang Li

**Affiliations:** 1 Department of Computer Science, Kennesaw State University, Marietta, GA 30060, USA; 2 Department of Mathematics, Stanford University, Stanford, CA 94305, USA; 3 Department of Electrical & Computer Engineering, Old Dominion University, Norfolk, VA 23529, USA; 4 Department of Ocean and Earth Sciences, Old Dominion University, Norfolk, VA 23529, USA; 5 Office of Research and Development, U.S. Environmental Protection Agency, Durham, NC 27711, USA

**Keywords:** bathymetry, machine learning, multi-spectral images, gradient boosting, CatBoost

## Abstract

Traditional bathymetry measures require a large number of human hours, and many bathymetry records are obsolete or missing. Automated measures of bathymetry would reduce costs and increase accessibility for research and applications. In this paper, we optimized a recent machine learning model, named CatBoostOpt, to estimate bathymetry based on high-resolution WorldView-2 (WV-2) multi-spectral optical satellite images. Cat-BoostOpt was demonstrated across the Florida Big Bend coastline, where the model learned correlations between in situ sound Navigation and Ranging (Sonar) bathymetry measurements and the corresponding multi-spectral reflectance values in WV-2 images to map bathymetry. We evaluated three different feature transformations as inputs for bathymetry estimation, including raw reflectance, log-linear, and log-ratio transforms of the raw reflectance value in WV-2 images. In addition, we investigated the contribution of each spectral band and found that utilizing all eight spectral bands in WV-2 images offers the best solution for handling complex water quality conditions. We compared CatBoostOpt with linear regression (LR), support vector machine (SVM), random forest (RF), AdaBoost, gradient boosting, and deep convolutional neural network (DCNN). CatBoostOpt with log-ratio transformed reflectance achieved the best performance with an average root mean square error (RMSE) of 0.34 and coefficient of determination (R^2^) of 0.87.

## 1. Introduction

Bathymetry, which measures the depth of the benthic floor in aquatic environments relative to the water level, defines the underwater topography of oceans, estuaries, and lakes [1]. It has applications in a wide range of fields, including navigation, benthic habitat mapping [2–4], the identification of marine objects in shallow waters such as seagrass and algae [5,6], sustainable management [7], hydrological modeling [8], monitoring sea-level rise due to climate change, and sediment removal [9–11]. These diverse use cases highlight the critical need for accurate and efficient bathymetric mapping. Historically, bathymetry was measured using a heavy rope, and creating a bathymetric map for a large region required significant manpower, equipment, time, and extensive in situ field observations. Although modern approaches such as airborne Light Detection and Ranging (LiDAR) [12] and Sound Navigation and Ranging (Sonar) systems [13,14] have been developed, each method still faces significant limitations. For example, the logistics and time required for vessel-mounted Sonar systems to cover an area, along with the need to correct for changing tidal states during data collection, restrict its use to ship-based operations. LiDAR is highly sensitive to water clarity and is associated with very high operational costs.

Recent technical advances have made remote mapping of bathymetry possible, and these technologies can be divided into two types: active and passive remote sensing [15]. Active sensors such as LiDAR have been widely employed for bathymetric mapping. LiDAR is capable of producing high-accuracy results in clear waters but is constrained by coarse spatial coverage and high operational costs [7]. In contrast, passive remote sensing techniques utilize reflected sunlight across multiple spectral bands to estimate water depth. Common multi-spectral platforms include SPOT [16], IRS-1C/1D and LISS-III [17], IKONOS [18], Landsat-8 [19], QuickBird [20], Sentinel-1 [21], Sentinel-2 [22], Worldview-2 (WV-2) [23], and hyperspectral imaging [24]. These methods offer extended depth detection capabilities compared to active methods, enabling bathymetric mapping in deeper waters, particularly in clear-water conditions. However, the accuracy of passive methods can be considerably affected by environmental factors, such as water clarity, turbidity, bottom reflectance characteristics, and atmospheric conditions, which collectively pose challenges to achieving reliable depth measurements [15,24].

Multi-spectral image-based methods assume that the depth of ocean is inversely proportional to the atmospherically corrected reflected energy from the water surface [15]. These methods can be categorized into two groups: (1) Traditional physics-based approaches, and (2) machine learning-based methods. For physics-based approaches, radiative transfer [17,23], linear transform [18], ratio transform [18,19], log-band ratio [20], optimal band ratio analysis (OBRA) [20], depth inversion limits of the FFT methodology [21], and downscaled coastal aerosol band [22] methods have been reported in the literature. With the growth of data availability and computational resources, machine learning models have gained increasing attention as powerful alternatives to physics-based approaches. Machine learning-based approaches can be categorized into three groups: (1) traditional models, such as linear regression (LR) [25–27], support vector machine (SVM) [28–30] and clustering [31], (2) ensemble-based methods including Bagging, least squares boosting, gradient boosting (GB) [32], and random forest (RF) [29,33,34], and (3) neural network and fuzzy logic systems such as artificial neural networks (ANN) [35], adaptive neuro-fuzzy inference systems [36], deep convolutional neural networks (DCNN) [37], deep variational autoencoders [38] and self-attention mechanisms [39].

Physics-based models often assume a straightforward linear relationship between surface reflectance and water depth, which fails to capture the intrinsic non-linearity of real-world aquatic environments [7]. This limits their ability to adapt to different conditions. Additionally, such models can be affected by factors like water clarity, bottom reflectance, and atmospheric conditions, leading to a decline in performance when faced with more complex environmental variables [15]. On the other hand, data-driven machine learning approaches are effective at capturing these non-linear relationships, but they have their own set of limitations. SVM is sensitive to feature selection and kernel parameters and often experiences decreased performance when dealing with high-dimensional remote sensing data [40]. RF models are robust but have limited generalization abilities when dealing with data with high noise levels or spatial heterogeneity, especially when sample sizes are small [41]. Deep learning methods like ANN and DCNN perform well with large datasets but usually require extensive labeled data for training [42]. Additionally, the generalization abilities of these models in bathymetric mapping are often limited due to spatial heterogeneity, resulting in limited spatial generalization across regions [43].

In the past few years, ensemble learning methods based on decision trees, especially Gradient Boosting, have significantly improved the accuracy of bathymetric estimation through stepwise optimization of weak learners [32]. Gradient boosting sequentially trains a set of weak decision tree learners by minimizing a differential loss function through gradient descent optimization. However, these boosting algorithms are prone to overfitting, especially with small or noisy datasets, because gradient calculations typically use the same training data as the models, lacking exposure to independent data for effective generalization [44]. To address these challenges, the Categorical Boosting (CatBoost) algorithm has been developed and applied for remote sensing bathymetric estimation [45]. The study in [46], which used multi-temporal Sentinel-2 imagery, showed that CatBoost significantly outperformed traditional LR models for bathymetry change estimation. Additionally, the results in [47] indicated that CatBoost could more accurately capture non-linear relationships compared to quasi-empirical models. However, both studies focused solely on Sentinel-2 images and lacked a comprehensive comparison with other machine learning methods. In this study, we explored the applicability of WV-2 images for bathymetry estimation under complex environmental conditions. We optimized the CatBoost model and compared its performance to state-of-the-art machine learning algorithms using datasets collected from three locations in Florida. In addition, we analyzed the contribution of individual spectral bands and found that utilizing full spectral range likely offers the best solution for handling complex water quality conditions, particularly when organic matter is present in the water.

## 2. Method

We collected water depth measurements using Sonar, along with corresponding multi-spectral satellite images across the Florida Big Bend region. We trained machine learning models to identify relationships between Sonar measurements and the pixel values in the multi-spectral images. After training, we used these models to estimate bathymetry for the entire study area from satellite imagery. Finally, we compared our bathymetry estimates with data provided by the National Oceanic and Atmospheric Administration (NOAA) and results from competing machine-learning models to evaluate performance.

### 2.1. Study Sites, Multi-Spectral Images, and Sonar Measurements

**Figure 1.**
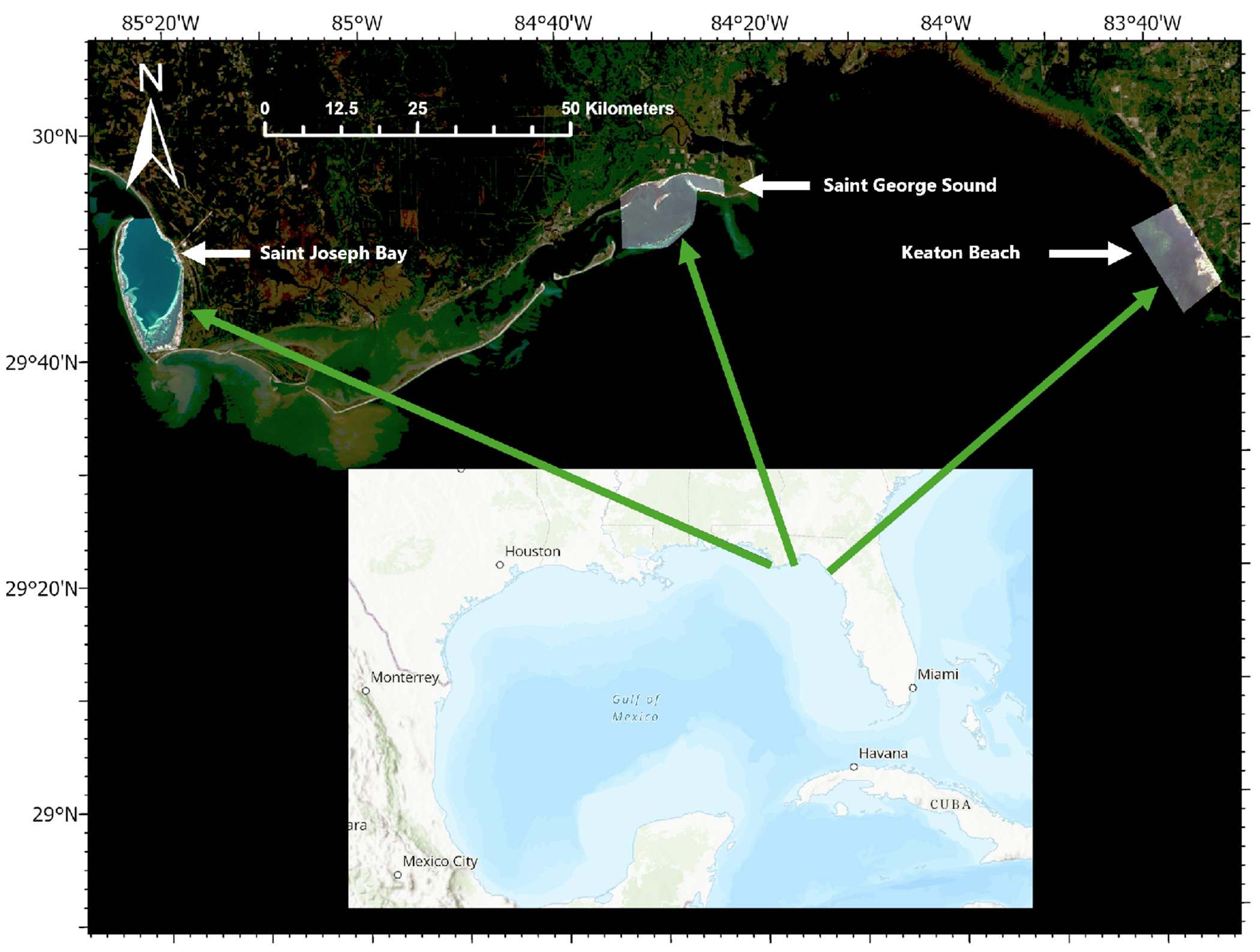
Study area in Mercator projection showing the three regions along the Florida Gulf Coast, USA, and the corresponding WorldView-2 satellite images analyzed in this study. From left to right: Saint Joseph Bay, Saint George Sound, and Keaton Beach. Imagery sources: CONANP, Esri, Garmin, FAO, NOAA, USGS, EPA, and Earthstar Geographics. Courtesy of [48].

We conducted our study at three sites along the Florida Gulf Coast, USA, including Saint Joseph Bay (SJB), Saint George Sound (SGS), and Keaton Beach (KB), as shown in Figure 1. The maximum water depth at SJB is approximately 9 m, at SGS is about 6 m, and at KB is about 5 m, as determined by the sonar measurements. Comprehensive measurements of water column optical properties and assessments of seagrass abundance were carried out at multiple stations within each study site [48]. These surveys and Sonar measurements collection were conducted near Keaton Beach between 18 and 26 May 2010, in Saint Joseph Bay from 1 to 9 November 2010, and in Saint George Sound between 24 April and 5 May 2012. The acquisition of WorldView-2 (WV2) imagery closely coincided with these field campaigns, with image dates being 20 May 2010 for KB, 14 November 2010 for SJB, and 27 April 2012 for SGS, ensuring minimal environmental variation between field and satellite observations. Saint Joseph Bay is unique among embayments in the eastern Gulf of Mexico, as it is not directly influenced by riverine input. However, it receives water enriched with colored dissolved organic matter (CDOM) from the Gulf County Canal, a human-made channel constructed in 1938 that connects the Intracoastal Waterway to the bay [49]. Saint George Sound forms the eastern part of the Apalachicola Bay estuary and lagoon system, an area heavily influenced by freshwater discharge from the Apalachicola River. This river drains a 50,000 km^2^ basin—the Apalachicola–Flint–Chattahoochee River Basin—which receives runoff from agricultural, industrial, and municipal sources. The coastal waters of Keaton Beach are influenced by CDOM-rich, oligotrophic discharge from the Steinhatchee River—a relatively short river that flows through a largely undeveloped watershed dominated by cypress swamps, titi swamps, peat bogs [50], and other diffuse sources along this section of the coastline.

**Figure 2.**
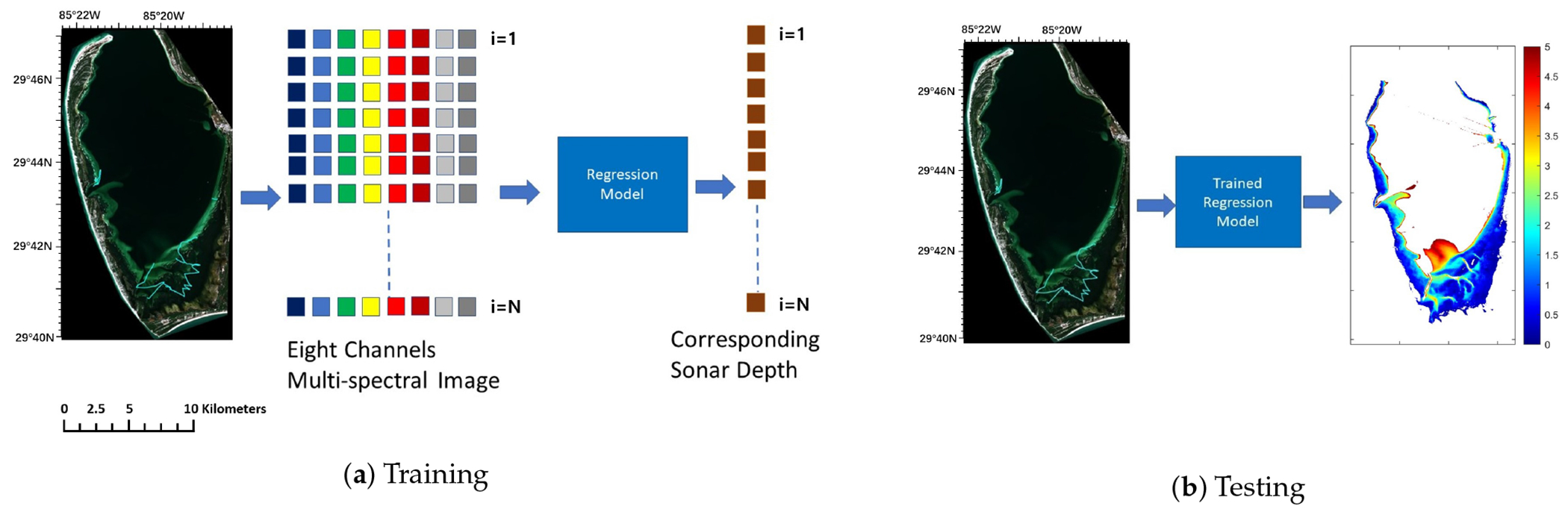
Proposed bathymetry estimation model (**a**) Training phase where *n* is the index of image patches (**b**) Testing phase. The collected Sonar measurements are shown in the image colored as cyan.

We utilized multi-spectral satellite images captured by the WV-2 satellite (operated by Maxar, Inc., Westminster, CO, USA) at the three study sites, and an example WV-2 image of SJB is shown in Figure 2. WV-2 provides eight spectral bands, including coastal-blue, blue, green, yellow, red, red-edge, near-infrared (NIR)-1, and NIR-2, with a spatial resolution ranging from 2 m to 3 m, depending on the sensor ’s view angle and imaging conditions. The spectral characteristics of these bands are listed in Table 1. At each site, downwelling spectral irradiance and upwelling spectral radiance were measured using two hyperspectral radiometer systems operating in tandem on a survey boat, both beneath and above the sea surface, resulting in a total of 22 in situ measurements across the three study sites. From these measurements, the spectral upwelling diffuse attenuation coefficient, upwelling radiance, and remote sensing reflectance were derived. The hyperspectral resolution of the field measurements was spectrally degraded to match the eight multi-spectral bands of the WV-2 sensor. A LR model was then performed between the 22 in situ measurements and the corresponding WV-2 spectra at each pixel to estimate the gain and offset for each band. These computed gain and offset values were subsequently applied to generate atmospherically corrected reflectances for the images [48,51].

In situ multibeam Sonar bathymetry data were recorded along ship tracks throughout the shallow regions of the study sites using a Humminbird 9800 multibeam chart plotter with internal GPS. Each sounding was time- and position-stamped by the GPS/chart plotter and corrected to mean lower low water (MLLW) by removing tidal effects using NOAA benchmark sheets and predicted tides [52]. Soundings were also corrected for the deployment depth of each transducer below the sea surface (10 cm). Figure 2 shows the Sonar tracks at SJB as blue lines. We collected 1635 Sonar bathymetry measurements at SJB, 13,096 measurements at KB, and 9189 measurements at SGS. A tidal correction of 2.2 m was applied to the measurements at SJB and KB, and 0.37 m at SGS, respectively, to match the tidal height at the time of the satellite pass. Multibeam Sonar bathymetry operates by emitting acoustic pulses towards the seabed and measuring the time taken for these pulses to reflect back to the transducer.

### 2.2. System Overview

The diagram of our proposed bathymetry model is shown in Figure 2. We first extracted a patch from the multi-spectral image and concatenated the pixels as an one-dimensional vector. During training, the CatBoost model [45] learned to fit the Sonar measurement at the location of the center pixel in an image patch based on the concatenated vector, as shown in Figure 2a. The parameters associated with CatBoost were optimized by the grid search through cross-validation (CV) and we named the optimized model as CatBoostOpt. After training, we applied the trained CatBoostOpt model to the whole multi-spectral image data to estimate bathymetry as shown in Figure 2b. We also investigated the contribution of each band to the estimation and implemented different models for comparison including linear regression [53], random forest [54], gradient boosting [55], ada-boost [56], SVM [57] and DCNN [58].

### 2.3. Proposed CatBoostOpt Model

Denote the collected dataset as $D={\left\{x_{i},y_{i}\right\}}_{i=1}^{N}$, where $x_{i}\in R^{M}$ is the *i*th input vector from a multi-spectral image patch, $y_{i}\in R$ is the depth value measured by Sonar corresponding to the center pixel in the patch, and *i* = 1, 2 … *N*. The CatBoost model [45] builds a sequence of oblivious trees [45] to approximate bathymetry *y* as $\overset{ˆ}{y}=F^{t}(x)$, where $F^{t}$ is built sequentially as $F^{t}=F^{t-1}+\alpha f^{t}$, and α is the step size, t=0,1,… represents the regression tree series. The base tree $f^{t}$ is obtained by minimizing the mean square error cost function:

(1) $$E(w)=\frac{1}{N}\sum_{i=1}^{N}{\left\{y_{i}-{\hat{y}}_{i}\right\}}^{2}=\frac{1}{N}\sum_{i=1}^{N}{\left\{y_{i}-\left[F^{t-1}\left(x_{i}\right)+f^{t}\left(x_{i}\right)\right]\right\}}^{2},$$

where **w** are the weights associated with the regression tree model.

To minimize the loss function in Equation (1), the CatBoost model applies a second-order approximation of the gradient *E*(**w**) with respect to $f^{t}$ at $F^{t-1}$. Estimating the gradient for Equation (1) using training data can lead to a prediction shift on unseen test data because $f^{t}$ is constructed in a greedy manner, where the residual errors modeled by $f^{t}$ are optimized on the same training data [45]. To address this issue, CatBoost employs a mechanism called ordered boosting, which ensures that data samples are ordered during training such that the data used to optimize $f^{t}$ are not utilized for obtaining $F^{t-1}$ [45].

There are several hyperparameters associated with the CatBoost model. To optimize these hyperparameters, we used a grid search through cross-validation during the model training process. This allowed us to automatically search for the optimal parameters that achieved the best performance with the training data. In the grid search, hyperparameters were defined on a grid. Each node on the grid was tested and the best node was chosen using the cost minimization function. Finally, the optimized hyperparameters were chosen for the model. The hyperparameters of CatBoost include:

- Learning rate: In CatBoost training, the model was updated by gradients of the predefined loss function, and the learning rate controlled the step size of the updates. A large learning rate leads to fast convergence but with a risk of an unstable solution. A small learning rate results in more stable solutions with slower convergence.
- Depth: The maximum number of layers or leaves in the decision tree of CatBoost to build. The capacity of CatBoost increases with the depth of the trees, but it causes the model to be overfitted to the training data.
- Iterations: Number of trees in the CatBoost model. Building more trees can increase the capacity but may also overfit the training data.
- Random strength: This refers to the degree of randomness used by each decision tree to split data [45].
- Bagging temperature: It refers to the usage of different distributions for the Bayesian bootstrap step on data to randomly assign weights. Larger values indicate more aggressive bagging.
- Border count: It controls the number of splits for each tree. Large values result in narrower trees. Optimizing this parameter affects training time and learning quality.

### 2.4. Log-Linear and Log-Ratio Transformations

The log-linear and log-ratio feature transformations of multi-spectral reflectance data have been shown to improve bathymetry estimation [18,31,59,60]. We compared performances of the machine learning models with and without these two transformations in this study.

Log-linear transform: This algorithm assumes that there is a log-linear relationship between multi-spectral reflectance data and the bathymetry values [18,31]. It uses a multiple linear regression method to predict bathymetry value *y* using reflectance values **x** as,

(2) $$\hat{y}=\sum_{j=1}^{M}w(j)log(x(j))+c,$$

where $\overset{ˆ}{y}$ is the predicted bathymetry value, x(j) is the reflectance value of the *j*th spectral band, w(j) is the weight associated with the *j*th spectral band, and *c* is the bias term. After log transforming of image reflectance, we estimate the linear relationship using the Scikit-learn toolbox [61] in Python.

Log-ratio transform: The log-linear model may not account for the unique properties of different sea bottom types, such as grass or sand, which can affect the reflectance values. To address this limitation, the log-ratio model [18,31] estimates the bathymetry value *y* based on the ratio of the reflectance values x(j) and x(k) from two different bands *j* and *k* as,

(3) $$\hat{y}=\sum_{j=1}^{M}\sum_{k=1}^{M}w(j,k)\frac{\log(x(j))}{\log(x(k))}+c.$$

We extracted a total of 56 features from the eight-band multi-spectral images using the log-ratio model, where j≠k and *M* is the number of bands in the image. The log-transformed reflectance values obtained from these features were then used to estimate the bathymetry.

### 2.5. Competing Models

#### Linear regression:

Linear regression estimates bathymetry *y* using a linear model based on independent multi-spectral reflectance data **x** as,

(4) $$\hat{y}=w^{T}x,$$

where **w** is the parameters of the linear model. This is a straight line in the input space and the linear regression method finds the optimal values for **w** that best define the linear relationship between bathymetry and image data. Best fit line is found by minimizing the mean square error cost function,

(5) $$E(w)=\frac{1}{N}\sum_{i=1}^{N}{\left(y_{i}-{\hat{y}}_{i}\right)}^{2}=\frac{1}{N}\sum_{i=1}^{N}{\left(y_{i}-w^{T}x_{i}\right)}^{2}.$$

We used the Scikit-learn [61] library to implement this model.

#### Support Vector Machine:

This model finds an optimal hyperplane between two classes to separate them with the largest margins [57]. SVM can use linear, polynomial, Gaussian, radial basis function (RBF), or sigmoid functions as kernels to achieve linear or nonlinear classification. SVM has also been extended to handle regression problems. In our study, we compared two SVM implementations for bathymetry estimation performance comparison: the fast implementation named *LinearSVM* and the popular implementation called *LibSVM* [62]. Both implementations are from the *Scikit-learn* library [61]. For *LibSVM*, we used an RBF kernel, which was relatively slower than the *LinearSVM* implementation.

#### Random Forest:

RF is an ensemble machine learning method consisting of many simple decision trees [54]. A decision tree separates data in feature space to perform classification or regression. RF builds an ensemble of decision trees by successively splitting data along the variable that minimizes the remaining variance in the response variable at each split. Each split within each tree considers only a random subset of the predictor variables, thus decorrelating trees within a random forest and minimizing issues presented by correlation among predictor variables [63]. Each decision tree generates its prediction to form the final decision by averaging. In addition, RF calculates importance of each feature. We used the *Scikit-learn* [61] library to implement this model.

#### Ada-boost:

Ada-boost is an adaptive ensemble machine learning method that trains a set of weak classifiers sequentially, with later classifiers focusing more on previously incorrectly classified data samples [56]. These weak classifiers are only slightly better than random guessing. The final model is a combination of all models weighted according to their performances. Ada-boost can also be extended to handle regression problems [64]. We utilized *Scikit-learn* to implement this model.

#### Gradient boosting:

GB is a machine learning method that trains a set of decision trees in a gradual and sequential manner [33]. It evaluates the training performances of models with current coefficients using the loss function and utilizes gradients to update and improve the models. The final model is a weighted ensemble of all trained models. We implemented the GB model using the *Scikit-learn* library.

#### Deep Convolutional Neural Network:

**Figure 3.**
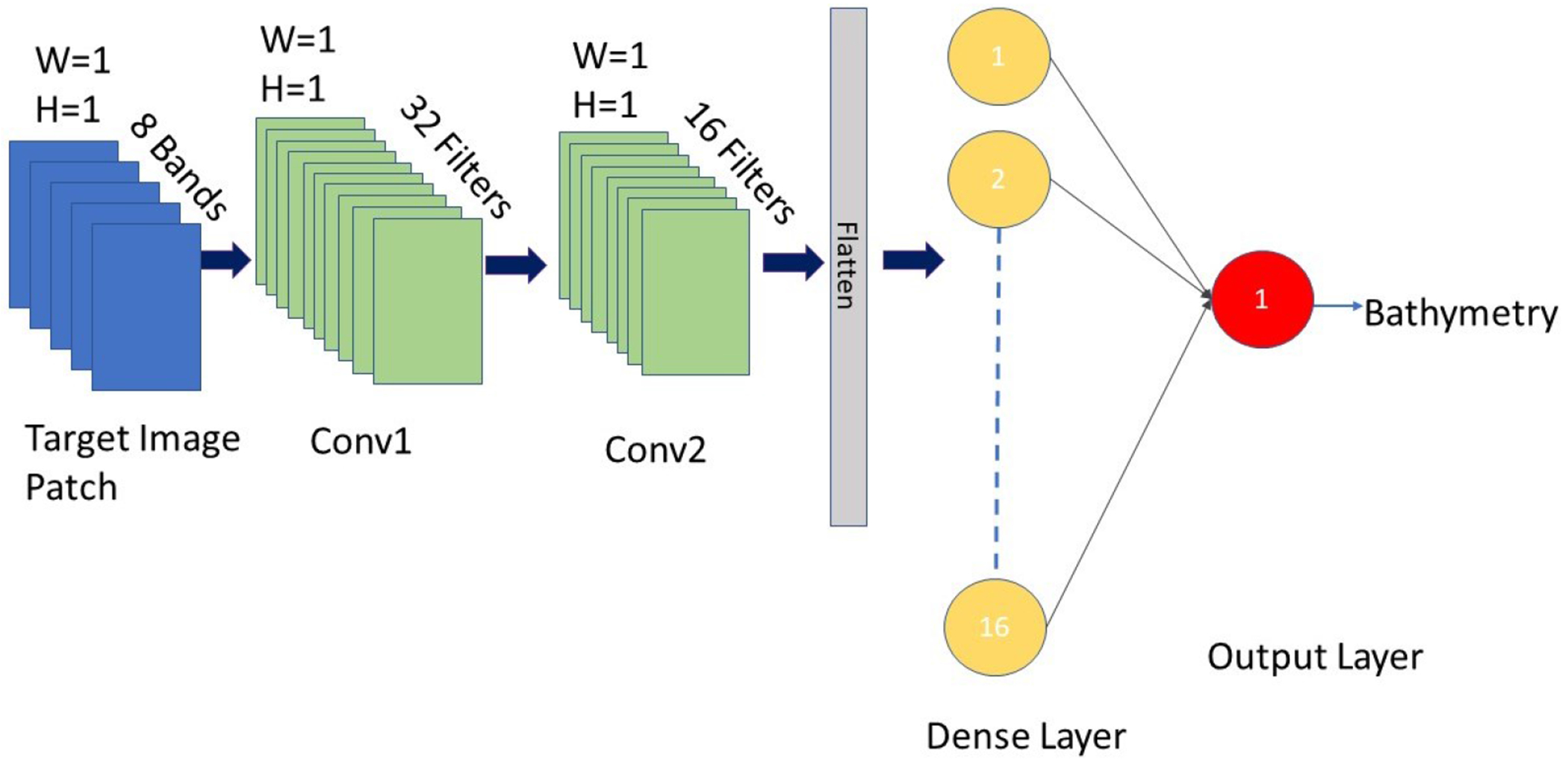
Bathymetry model using DCNN, where *H* and *W* represents height and width, *conv1* and *conv2* represents convolution layers 1 and 2.

DCNN achieved state-of-the-art results in image classification [58], image segmentation [65], and object detection. Previously, we used a DCNN model for seagrass detection [66,67]. We use a similar architecture for bathymetry quantification as shown in Figure 3. The proposed DCNN regression model contains six layers: two convolutional layers, two dropout layers, one flatten layer, and one regression layer. The first and second convolutional layers have 32 and 16 convolutional kernels with a size of 1 × 1 pixels, respectively. Each convolutional layer is followed by a dropout layer with a probability of 0.01 to reduce over-fitting. In the last layer, we use a fully connected layer with 16 hidden units to predict the bathymetry values. We use 1 × 1 × 8 image patches as inputs for the DCNN model to predict bathymetry information.

### 2.6. Evaluation Metrics

We utilized multiple evaluation metrics, including root mean square error (RMSE), RMSE percentage (RMSE (%)), mean absolute error (MAE), mean squared error (MSE), mean absolute percentage error (MAPE), and the coefficient of determination (R^2^) to assess the accuracy of different bathymetry estimation models against in situ measured bathymetry. The R^2^ metric quantifies the proportion of variance in the dependent variable that is explained by the independent variables, with a perfect score of 1.0 indicating a perfect fit. RMSE, MAE, MSE, RMSE (%), and MAPE (%) are non-negative, with lower values indicating better model performances. The evaluation metrics are defined as follows:

(6) $$\text{MSE}=\frac{1}{N}\sum_{i=1}^{N}{\left(\hat{y}-y\right)}^{2},$$

(7) $$\text{MAE}=\frac{1}{N}\sum_{i=1}^{N}|\hat{y}-y|,$$

(8) $$\text{RMSE}=\sqrt{\frac{1}{N}\sum_{i=1}^{N}{\left(\hat{y}-y\right)}^{2}},$$

(9) $$\text{RMSE}(\text{\%})=\left(\frac{\text{RMSE}}{\bar{y}}\right)\times 100,$$

(10) $${\text{R}}^{2}=1-\frac{\sum_{i=1}^{N}{\left(\hat{y}-y\right)}^{2}}{\sum_{i=1}^{N}{\left(y-\bar{y}\right)}^{2}},$$

(11) $$\text{MAPE}(\text{\%})=\frac{1}{N}\sum_{i=1}^{N}\left|\frac{\hat{y}-y}{y}\right|\times 100,$$

where *y* represents the sonar-measured bathymetry value, $\overset{ˆ}{y}$ denotes the model-predicted bathymetry value, $\overset{‾}{y}$ is the mean of the observed bathymetry values, and *N* is the number of data samples.

## 3. Experiment Setup

We conducted four experiments in this study to investigate the effectiveness of using multi-spectral images combined with the CatBoostOpt model for estimating bathymetry in shallow sea areas.

### Experiment 1: Hyperparameter determination.

The bathymetry estimation models have many hyperparameters, some of them related to the machine learning model and others are related to how we organize the data or how we train the models, e.g., the size of image patches extracted from multi-spectral images for bathymetry. In the CatBoost model, there are seven hyperparameters in the model as listed in Section 2.3. We utilized trial and error to optimize these hyperparameters based on grid search and selected a set of hyperparameters that best fitted our datasets for bathymetry.

### Experiment 2: Band importance investigation.

To evaluate the impact of each image band on model performance, we conducted a series of experiments to quantify the contribution of each spectral band and to compare the effectiveness of different band combination strategies across three locations using the CatBoostOpt model.

### Experiment 3: Comparison study for bathymetry estimation.

We compared the Cat-BoostOpt model with other machine learning approaches for baythymetry estimation through 3-fold CV on the collected Sonar measurements and corresponding multi-spectral images as described below,

- We extracted image patches (**x**) from the multi-spectral images at the locations where Sonar measurements (*y*) were taken, and created a dataset $D=xi,{y_{i}}^{N}i=1$, where **x** and *y* represent an image patch and Sonar measurement, respectively.
- We performed 3-fold CV on the dataset and evaluated the performance of each model in terms of MAE, RMSE, MSE, RMSE(%), MAPE(%), and R^2^.
- We repeated the above steps at all three locations including SJB, KB, and SGS.

### Experiment 4: Case study for bathymetry estimation.

We trained all the competing models with all the Sonar measurements and applied the trained model to multi-spectral images collected at the three sites to estimate bathymetry. The estimated bathymetry maps were then compared with the historical bathymetry data collected by the NOAA.

## 4. Results

### 4.1. Experiment 1: Hyperparameter Determination

#### Cross-validation setting:

It is common to utilize *k*-fold CV for model performance assessment. To determine an appropriate value for *k*, we conducted 3-, 5-, and 10-fold CV experiments using the CatBoostOpt model on the Keaton Beach dataset as shown in Table 2. The results indicated that increasing the number of folds did not lead to significant differences in model performance. Given that the differences in key evaluation metrics (e.g., RMSE, MAPE, and R^2^) were marginal across different *k* values, we selected 3-fold CV for the subsequent experiments, as it provides a good balance between computational efficiency and model generalization. This choice allows for a substantial reduction in training time while maintaining comparable predictive accuracy.

#### Image patch size determination:

To determine a suitable patch size for bathymetry estimation, we compared 3-fold CV results of different image patch sizes of 1 × 1 × 8, 3 × 3 × 8, and 5 × 5 × 8 with CatBoost on SJB data and results are shown in Table 3. It was observed that model performance did not significantly improve with larger patch sizes. Using a 1 × 1 patch size limits the capture of spatial texture information but offers key advantages for bathymetric estimation. It preserves a direct pixel-level relationship between spectral features and depth, avoiding spatial mixing introduced by larger patches. The pixel-level approach also minimizes boundary effects, improving depth estimation near image edges. Additionally, it lowers input dimensionality and model complexity, which is beneficial for training on limited data. Given these benefits and the limited gains from larger patches, the 1 × 1 × 8 configuration was selected as optimal in this study.

The optimized hyperparameters for CatBoostOpt were determined as follows: iterations = 2000, depth = 10, learning rate = 0.01, *L*_2_ leaf regularization = 1, bagging temperature = 0, and border count = 255. These hyperparameters were selected based on extensive experimentation to achieve the best model performance given our specific dataset and application scenario. To assess the impact of hyperparameter optimization, we also compared the CatBoostOpt with the default CatBoost configuration in Experiment 3.

### 4.2. Experiment 2: Band Importance Investigation

#### Band importance:

**Figure 4.**
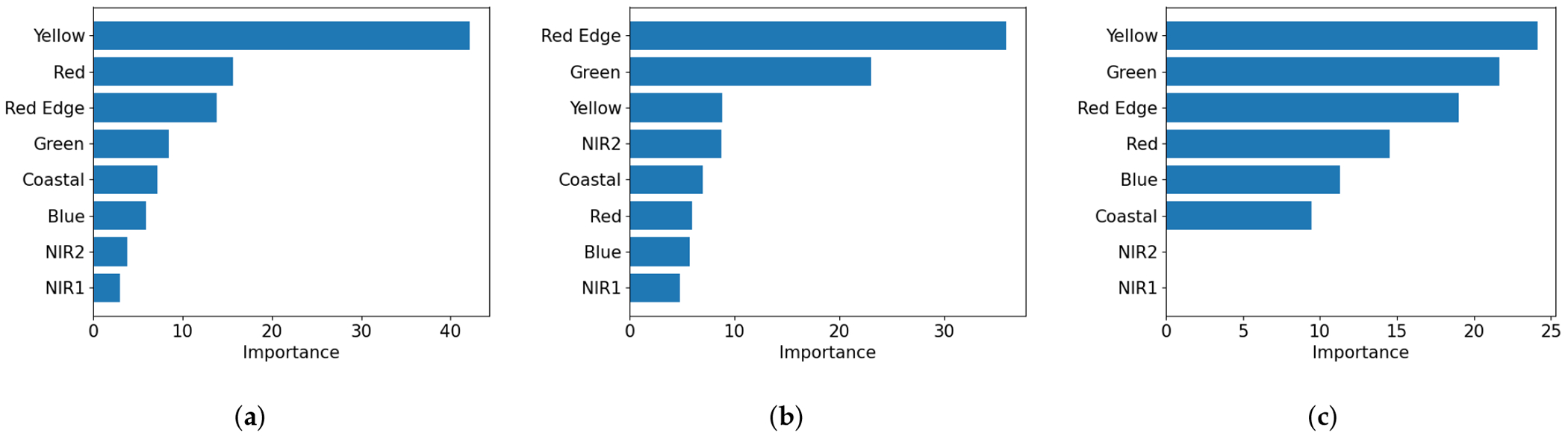
Band importances of the eight spectral bands in WV-2 images at (**a**) SJB (**b**) KB and (**c**) SGS.

During model training, the CatBoost model calculates an importance score for each input feature, indicating how much the prediction value changes (on average) when that feature is used in the model [45]. We listed the feature/band importance scores for all eight bands in the WV-2 image generated by the CatBoostOpt model using raw reflectance, as shown in Figure 4, across the three locations: KB, SJB, and SGS. It can be observed that different spectral bands exhibit varying importance rankings across the three regions. At the SJB location, band 4 achieved the highest importance score of 34.41%. In contrast, band 6 was the most significant at the KB location with a score of 35.19%. At the SGS location, band 4 ranked highest, but with a lower importance score of 23.51% compared to the other two locations.

**Figure 5.**
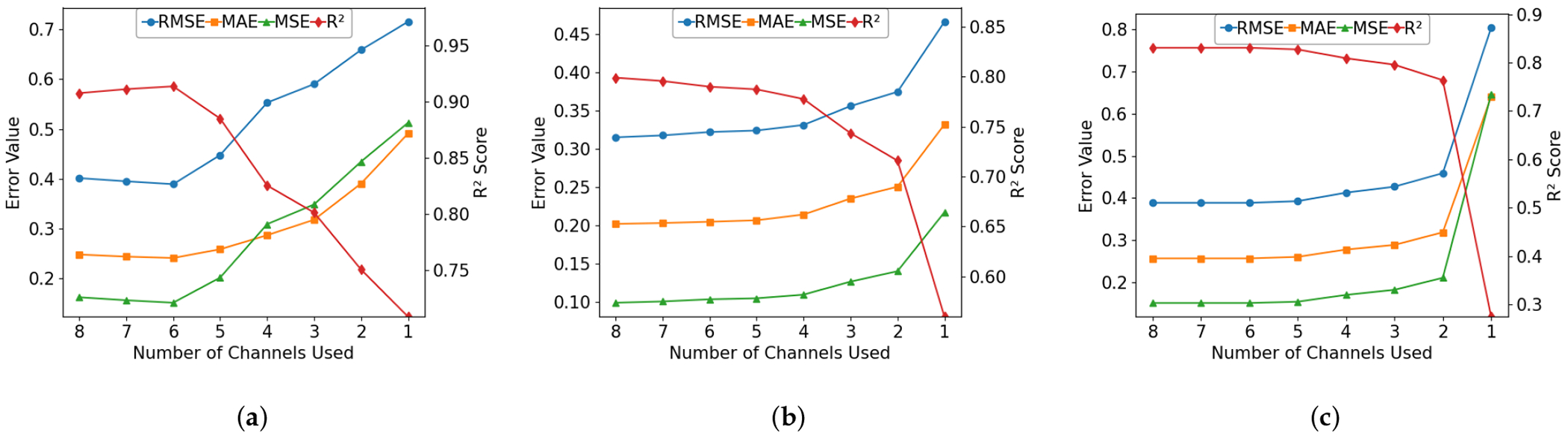
Performances of CatBoostOpt with progressively eliminating low-importance bands in WV-2 images at (**a**) SJB (**b**) KB and (**c**) SGS.

Based on the band importance scores at the three locations, we conducted band elimination experiments. We first utilized all bands in a 3-fold CV setting with the CatBoostOpt model, and subsequently eliminated one least important band at a time across all three locations. Figure 5 shows the performance of the experimental results, where the X-axis represents the number of bands used, the left Y-axis corresponds to *RMSE*, *MAE*, and *MSE*, and the right Y-axis represents *R*^2^. The results indicate that the model accuracy decreases significantly as bands are removed, reaching the worst performance when only a single band remains.

#### Band combination strategies:

Water effectively absorbs incoming light, causing the amount of light penetrating the ocean to decrease rapidly with depth (a process known as attenuation). Moreover, different wavelengths of light are absorbed at different rates, with shorter wavelengths penetrating deeper into the water column [68]. To evaluate the effectiveness of different spectral bands for bathymetry estimation, we conducted experiments using various band combinations as follows: (1) All eight spectral bands provided by the WV-2 sensor, (2) The top six bands ranked by feature importance scores, and (3) Four commonly used bands for bathymetry estimation, including Coastal band (400–450 nm), Blue band (450–510 nm), Green band (510–580 nnm) and Yellow band (585–625 nm).

Table 4 presents the results of the 3-fold CV test of the CatBoostOpt model using three different band combinations. The average results demonstrate that utilizing all eight spectral bands yields the most reliable and accurate predictions with CatBoostOpt across different locations. The configuration using the top six most important bands shows a slight decrease in performance. Conversely, the traditional selection of the standard four bands performs significantly worse across all metrics, suggesting that this strategy fails to adequately capture the full characterization of the data.

### 4.3. Experiment 3: Comparison Study

#### Results of 3-fold CV:

Tables 5–7 show 3-fold CV results by different models with raw reflectance, log-linear and log-ratio of raw reflectance as inputs, respectively, at the three Florida locations. It was observed that the default CatBoost model outperformed all other competing models. The optimized CatBoost model, CatBoostOpt, using log-ratio of raw reflectance in multi-spectral images as inputs further improved the performances at all the three locations, and performed the best in terms of all the performance metrics (Table 7).

#### Scatter plots of 3-fold CV results:

**Figure 6.**
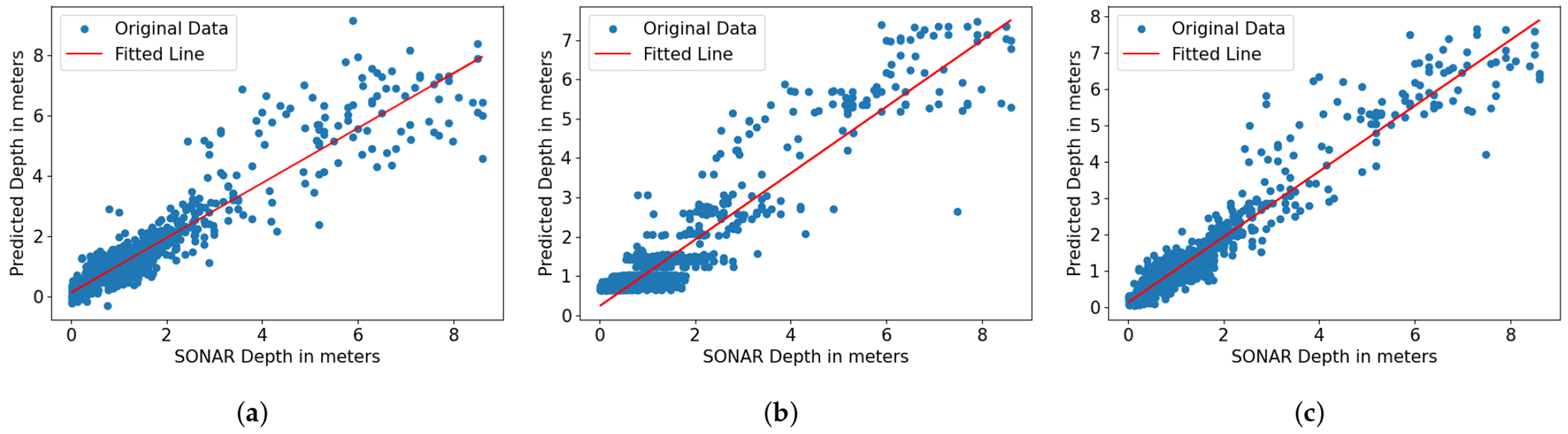

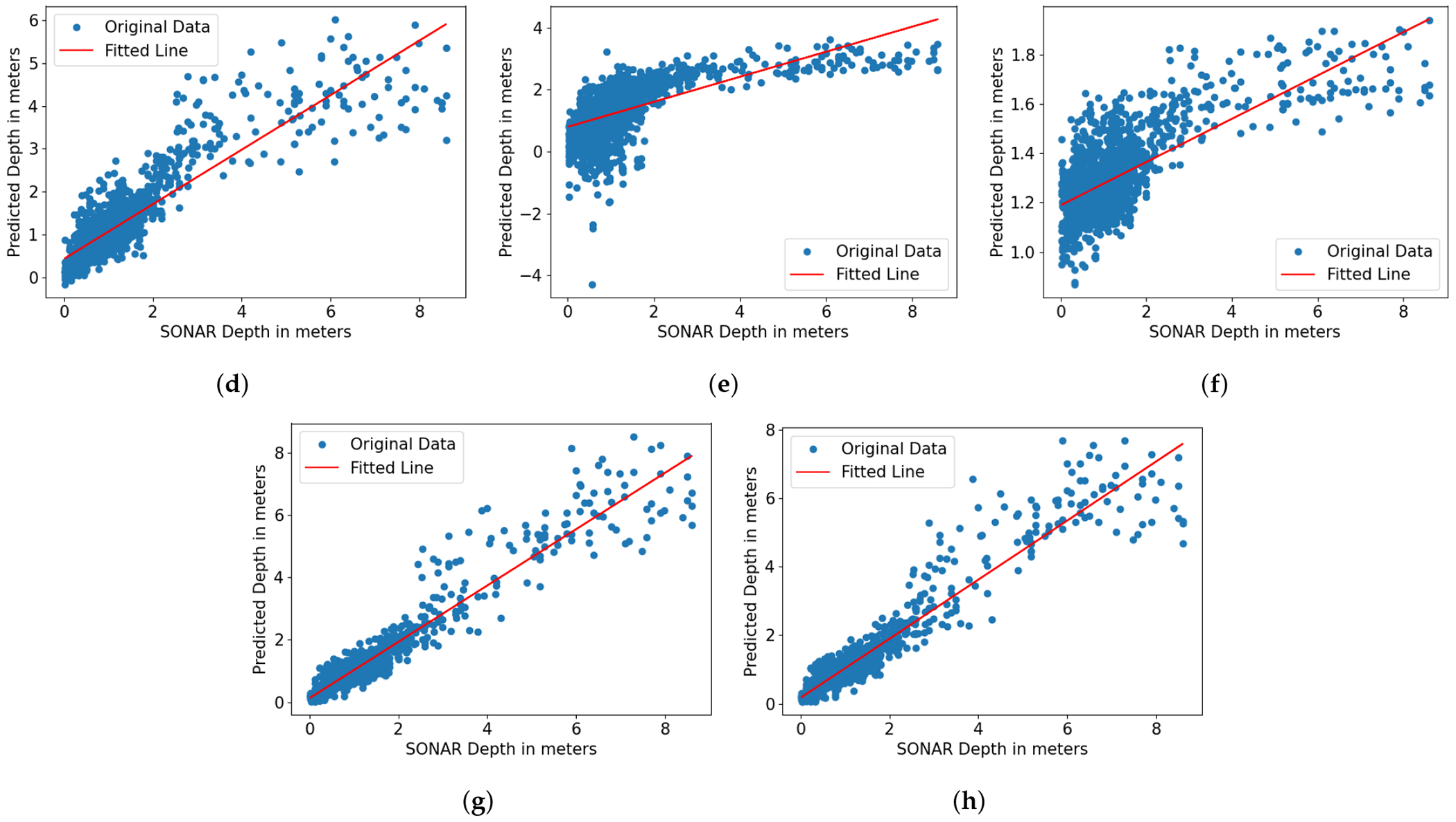
Scatter plotting of 3-fold CV results at SJB. (**a**) GB (**b**) AdaBoost (**c**) RF (**d**) Lib-SVM (**e**) LR (**f**) DCNN (**g**) CatBoost and (**h**) CatBoostOpt. X-axis represents sonar bathymetry depth and Y-axis represents machine learning model predicted bathymetry.

**Figure 7.**
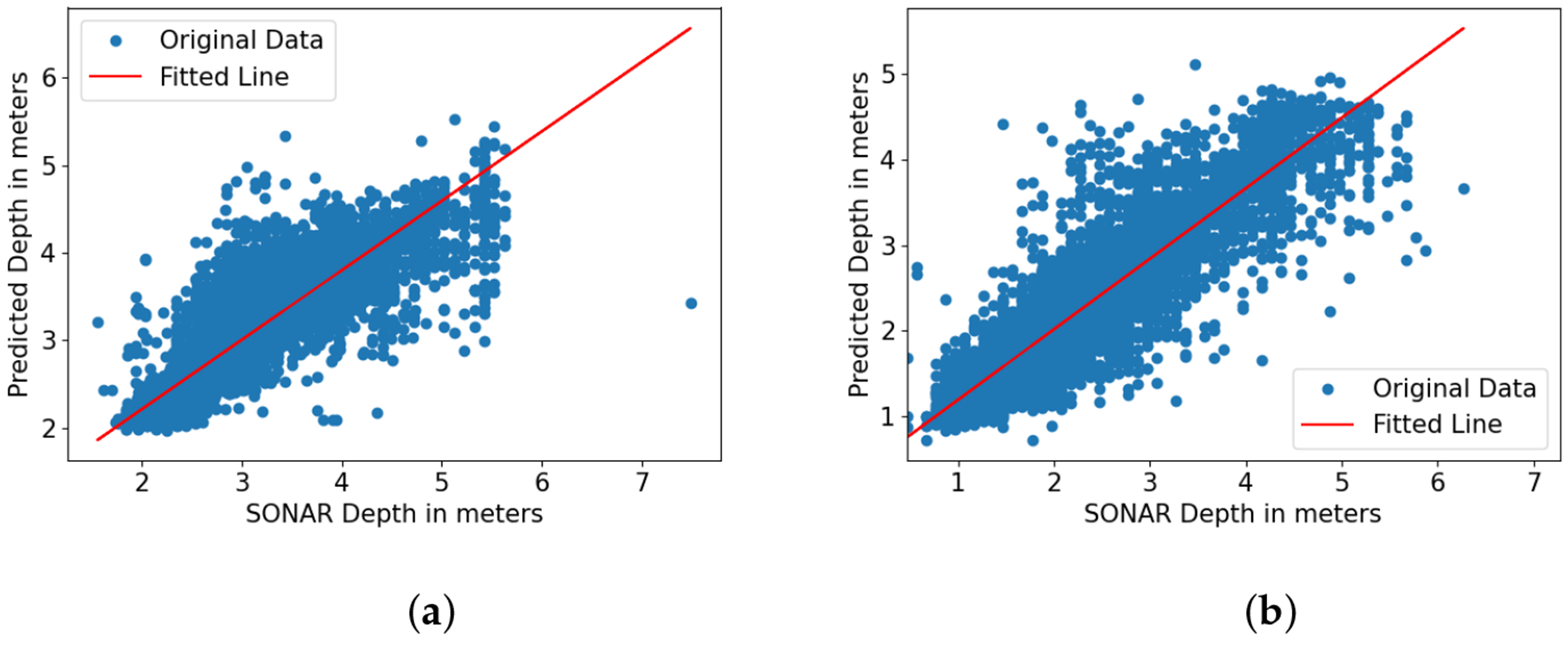
Scatter plotting of 3-fold CV results by CatBoostOpt at: (**a**) Keaton Beach and (**b**) Saint George Sound.

Figure 6 shows scatter plots of 3-fold CV results for different models at SJB. The *x*-axis represents bathymetry depths from Sonar measurements, while the *y*-axis represents bathymetry predicted by each model. The red line denotes the best-fit line, minimizing the distance from data points to the line. A slope closer to 1 indicates a better model fit. The proposed CatBoostOpt model outperformed all other models. The 3-fold CV results in Table 5 further confirm that CatBoostOpt achieved the best performance at KB and SGS. Scatter plots of CatBoostOpt at KB and SGS are shown in Figure 7, where the slopes of the best-fit lines remain close to one. We observe that the relationship between reflectance and Sonar bathymetry is distinctly non-linear, with saturation effects evident in the results produced by SVM, linear regression, and DCNN methods.

**Figure 8.**
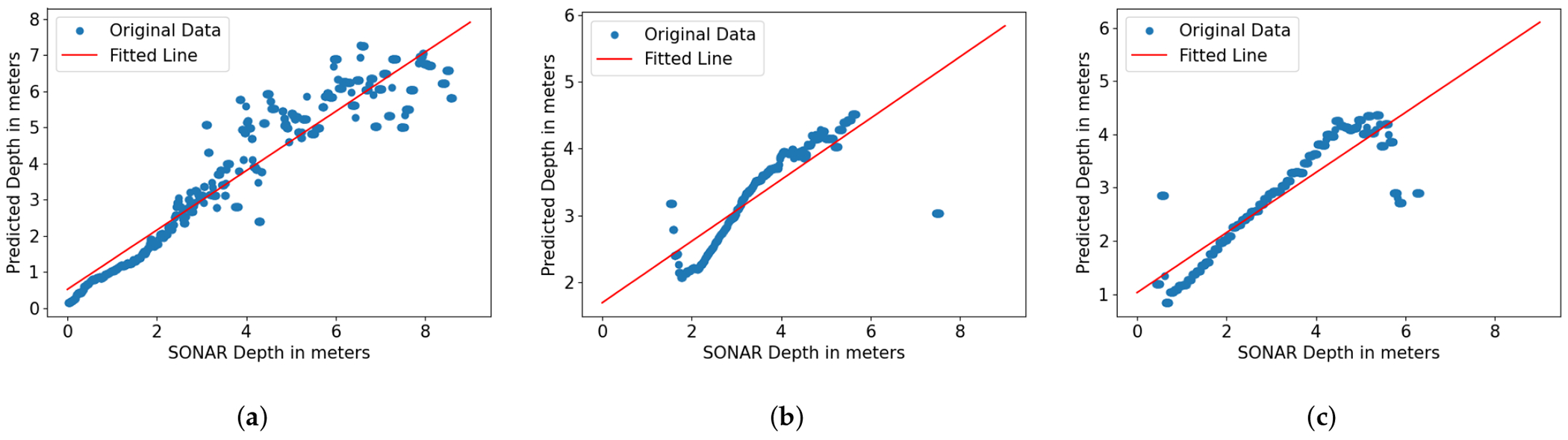
Scatter plotting of 3-fold CV results by CatBoostOpt regression at (**a**) SJB (**b**) KB and (**c**) SGS.

To avoid cluttered and dense scatter plots, we used average value scatter plotting at all three locations in Florida. We divided all available Sonar bathymetry data into bins with an interval of 0.01 m. We then averaged all Sonar bathymetry data and model predictions belonging to a particular bin and plotted the averaged prediction against Sonar data to present a clear representation of scatter plotting, as shown in Figure 8. It is observed that the CatBoostOpt model performed very well at locations where the bathymetry is less than 4 m.

### 4.4. Experiment 4: Case Study

**Figure 9.**
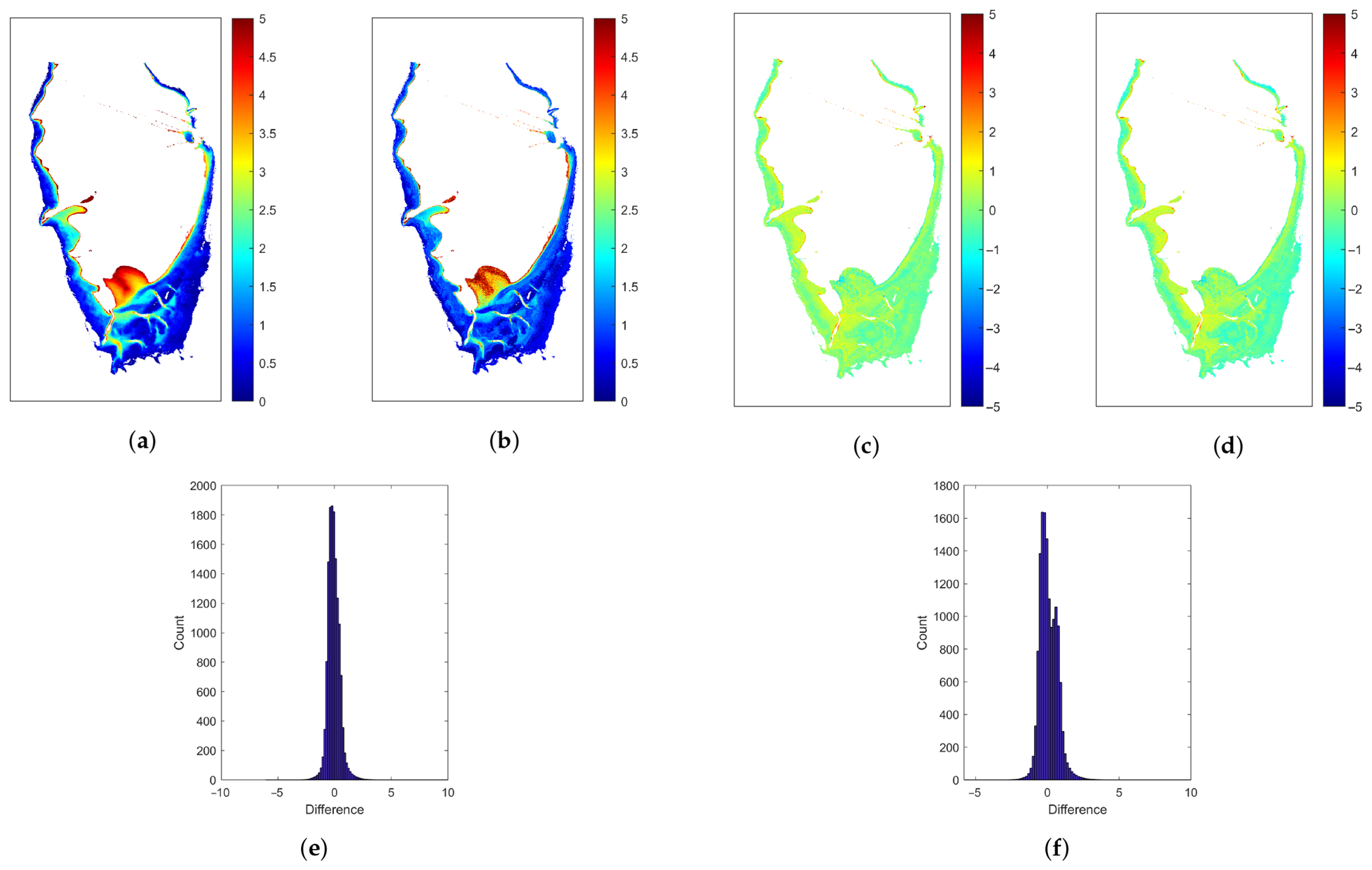
Bathymetry estimation results at SJB. (**a**) Bathymetry from NOAA, (**b**) Bathymetry by CatBoostOpt, (**c**) Difference between (**a**) and (**b**), (**d**) Difference in the seagrass regions, (**e**) Histogram of the difference between NOAA and predicted bathymetry, and (**f**) Histogram of the difference in seagrass regions. Unit is meter (m).

**Figure 10.**
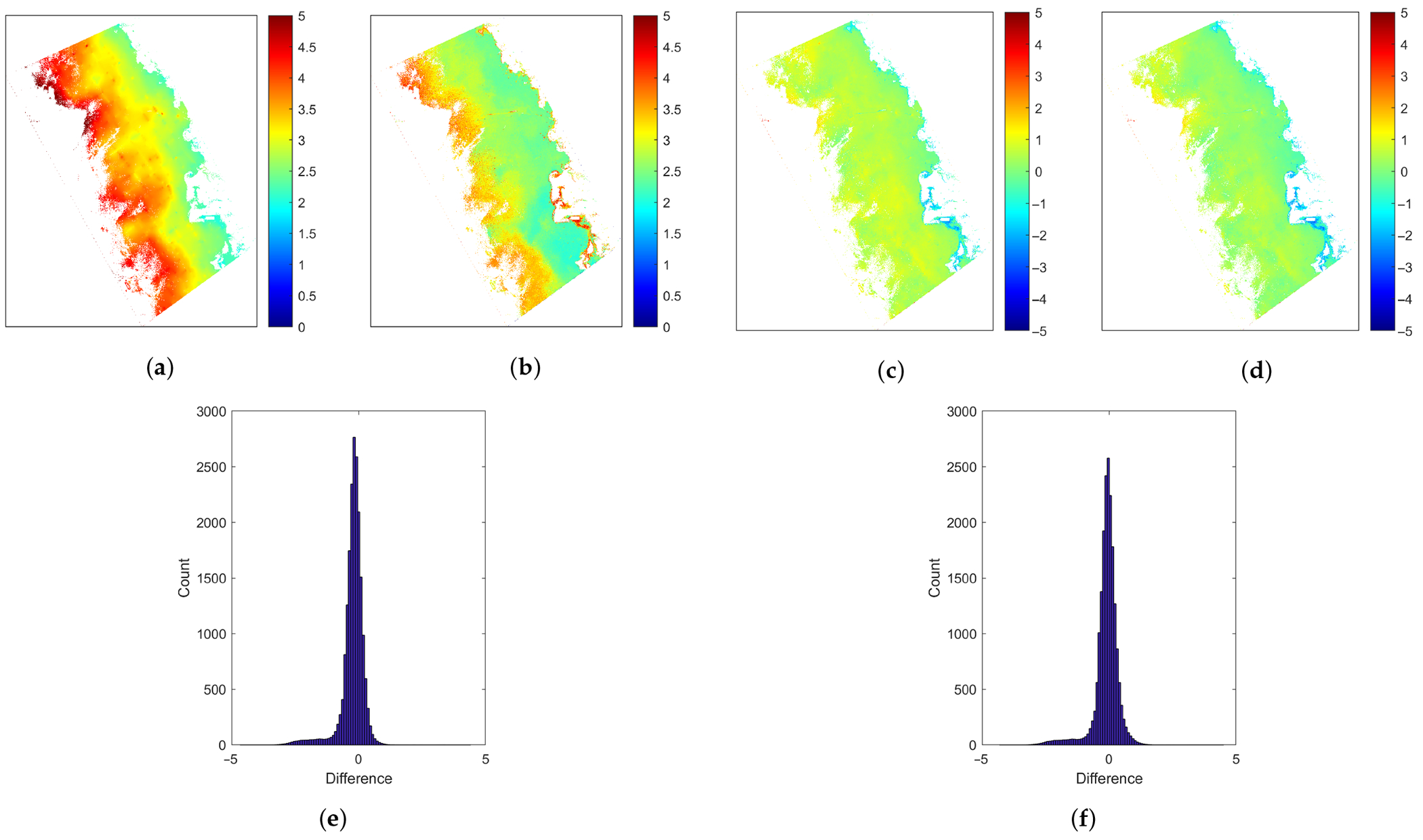
Bathymetry estimation results at KB. (**a**) Bathymetry from NOAA, (**b**) Bathymetry by CatBoostOpt, (**c**) Difference map between (**a**) and (**b**), (**d**) Difference map in the seagrass regions, (**e**) Histogram of the difference between NOAA and predicted bathymetry, and (**f**) Histogram of the difference in the seagrass regions.

**Figure 11.**
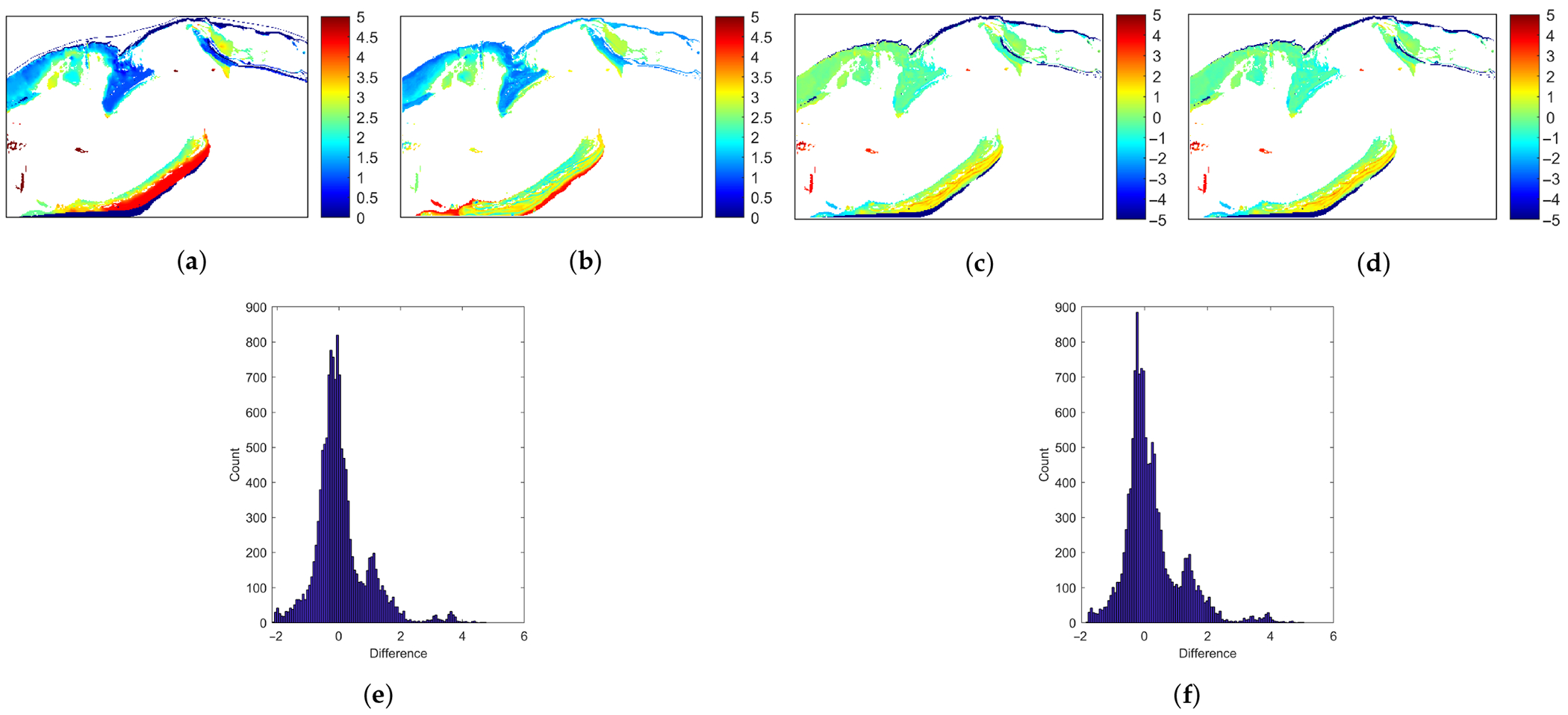
Bathymetry estimation results at SGS. (**a**) NOAA bathymetry, (**b**) CatBoostOpt predicted bathymetry, (**c**) Difference map between (**a**) and (**b**), (**d**) Difference map in seagrass regions, (**e**) Histogram of the difference between NOAA and predicted bathymetry, and (**f**) Histogram of the difference in seagrass regions.

We utilized all the Sonar measurements at one location to train the CatBoostOpt model and applied the trained model to the whole multi-spectral image for bathymetry estimation. The estimation maps for SJB, KB, and SGS are shown in Figure 9, Figure 10 and Figure 11, respectively. Using the model prediction map at SJB as an example (Figure 9b), it is observed that the differences between the model predicted bathymetry values and NOAA data are mostly around 0 as can be confirmed by the green color in Figure 9c and the histograms in Figure 9e. The differences in seagrass region (Figure 9d,f) are also mostly around 0. Satellite imagery usually fails to obtain correct bottom reflectance in areas where seagrass grows. In our study, we added a 0.3 m correction value to seagrass region for compensation. We followed the same procedure for KB and SGS locations, and results are shown in Figure 10 and Figure 11, respectively. RMSEs between NOAA data and model predictions are shown in Table 8 for the three locations. It can be observed that differences increased at KB after the seagrass correction was applied.

## 5. Discussion

Different multi-spectral remote sensing imagery and machine learning algorithms have been applied to estimate bathymetry in the literature. However, it is difficult to make a fair comparison due to the fact that each study utilized different image modalities, algorithms, and/or different locations. For example, Tonion et al. [29] achieved a mean average error of 0.158 m using a random forest algorithm with combined image modalities of Planetscope, Sentinel 2, and Landsat 8 satellite images in Cesenatico, Italy. Misra et al. [28] achieved an RMSE of 0.35 m using SVM with Landsat 7 ETM+ and Landsat 8 OLI image modalities in Ameland Inlet, Netherlands. Eugenio et al. [23] achieved RMSEs of 1.2 m and 1.94 m in the Granadilla and Corralejo areas, respectively, with Worldview-2 images. Our study provided a fair comparison platform for different machine learning algorithms in bathymetry estimation since all algorithms used the same dataset. Our results showed that the recent machine learning algorithm, CatBoost, when optimized, outperformed all competing machine learning algorithms including linear models, SVM, convolutional neural networks, and other boosting algorithm families. The optimized CatBoost algorithm, combined with the log-ratio transformation of WV-2 images, achieved RMSEs of 0.33 m, 0.29 m, and 0.37 m at SJB, KB, and SGS, respectively, a significant improvement compared to other algorithms.

Though WV-2 images are limited to retrieving depths of approximately 5 m, their high spatial resolution and rich multi-spectral information provide significant advantages for accurately estimating bathymetry in complex shallow-water environments, where detailed spatial variation is critical. An important consideration in our study is the emerging capability of satellite-based bathymetry, which has demonstrated the ability to capture smaller-scale details in bathymetry, such as sand ripples in regions like Saint George Sound. This enhanced spatial resolution surpasses that of traditional NOAA methods, providing a more detailed view of the seafloor. These finer-scale depth variations are particularly significant when mapping seagrass density and predicting seagrass presence or absence using depth-driven models. The ability to resolve these small-scale features can lead to more accurate and effective conservation and management strategies for seagrass habitats.

Gradient boosting utilizes a greedy algorithm to sequentially train an ensemble of decision trees to fit the data and outperformed all other non-boosting algorithms by large margins as shown in Tables 5–7. However, there remains a risk of overfitting, especially when dealing with small and noisy datasets. One of the major improvements of CatBoost over traditional gradient boosting is its use of separate, unseen data to compute the gradients of the loss function during training, which reduces the risk of overfitting when the available data are noisy or limited [45]. To further improve the CatBoost model, we optimized the model’s hyperparameters and compared the performance of the original CatBoost model with that of the optimized version, CatBoostOpt. The results obtained by CatBoostOpt showed significant improvement, indicating that hyperparameter tuning was essential for enhancing model performance. Without optimization, the model tended to underfit, particularly in complex coastal environments, thereby limiting its effectiveness for bathymetric estimation.

In bathymetry estimation, raw, log-linear, and log-ratio are commonly used transformations. The raw reflectance maintains the physical meaning of the measurements but often exhibits skewed distributions due to large magnitude differences and non-linear relationships among the measurements [31]. The log-linear transformation reduces the influence of large values and enhances sensitivity to small values, which is particularly important for shallow water bathymetry. However, the transformed values lose their direct physical interpretation [59]. The log-ratio transformation calculates the ratio between different bands to enhance depth-related information, effectively normalizing for water turbidity, bottom reflectance, and mitigating the influence of varying environmental conditions [18]. In our study, the raw and log-linear transformed reflectance performed similarly, as shown in Tables 5 and 6, while the log-ratio transformed reflectance achieved the best performance at all three locations, as shown in Table 7, showcasing the super performances of the log-ratio transformed reflectance for bathymetry.

Bathymetry estimation typically utilizes short-wavelength bands, such as Coastal, Blue, and Green, and sometimes also includes Yellow, since these wavelengths can penetrate water more deeply [68]. However, the study sites examined in this paper are influenced by CDOM-rich, oligotrophic discharge from terrestrial water sources [50], along with other diffuse sources along this section of the coastline. The presence of CDOM significantly alters the optical properties of the water, and our band importance analysis revealed that the contribution of each band to bathymetry estimation is not consistent with the basic physics discussed in [68], due to the influence of CDOM. Our band elimination study further demonstrated that using all eight bands available in the WV-2 sensor provided no substantial performance difference compared to using the top six bands. Therefore, we utilized all eight bands in our study to better mitigate the CDOM effects across different locations.

The DCNN was consistently underperformed, regardless of the reflectance transformation method employed. This underperformance may stem from the limited spatial structure in the input data, as only pixel-wise spectral features were available, which hampers the convolutional layers’ ability to capture meaningful spatial patterns. Additionally, DCNNs typically require large datasets for effective training, and the relatively small dataset used in this study may have led to overfitting and reduced generalization capability.

The bathymetry correction step in the seagrass area did not always reduce the overall error. The RMSE between the NOAA bathymetry and the model-predicted bathymetry with and without the correction is shown in Table 8. The correction step reduced RMSE errors at the KB location but increased RMSE errors at SJB and SGS. We obtained the best RMSE values of 0.50 and 0.87 without seagrass correction at SJB and SGS, respectively, and achieved the best RMSE of 0.52 at KB with the correction, as listed in Table 8. The varying effects of the seagrass correction across different sites may be attributed to differences in seagrass density, species composition, and local water clarity. Additionally, the correction algorithm assumes that seagrass has a uniform influence across all study sites. However, this may not accurately represent the spatial variability of the submerged vegetation.

Our study advances bathymetry estimation by improving upon the methodologies employed in previous research [69]. The CatBoost model achieved R^2^ values between 0.84 and 0.92 with RMSE below 0.5 m for depths shallower than 7 m, while CatBoostOpt further improved accuracy, achieving RMSE values of 0.36 m, 0.30 m, and 0.38 m at SJB, KB, and SGS, respectively. Unlike previous studies, we evaluated different reflectance transformations, demonstrating that log-ratio transformation significantly enhances depth estimation accuracy. Additionally, our study validated the model across multiple coastal locations in Florida, demonstrating its robustness under diverse environmental conditions. Overall, our findings confirm that hyperparameter tuning and proper reflectance transformations are critical for improving satellite-based bathymetric mapping, providing an accurate and scalable solution for coastal monitoring.

In this study, we still need to train a separate model for bathymetry estimation at different locations because the multi-spectral images collected at different locations are typically impacted by the quality of water at that location, making pixel values in the images location-dependent. We assumed that the depth of the ocean is inversely proportional to the atmospherically corrected reflected energy from the water surface. However, we did not consider the effects posed by the quality of water, which means that the same pixel values in the image may represent different ocean depths at different locations if the water quality varies. Potential solutions include training separate models for different locations if data are available, or adjusting models that were trained for different locations to a specific location by taking water quality into account. In the machine learning community, domain adaptation techniques [70] are also possible solutions for this challenge.

## 6. Conclusions

We developed an optimized CatBoost model for bathymetry estimation using multi-spectral satellite imagery. The model effectively captured the complex nonlinear relationships between Sonar-derived bathymetric measurements and spectral reflectance values, enabling accurate depth predictions. Validation at three locations in Florida demonstrated that the proposed model consistently outperformed other competing methods, achieving RMSEs of 0.36 m, 0.30 m, and 0.38 m at SJB, KB, and SGS against NOAA bathymetry data. These results highlight the model’s robustness in different coastal environments and its potential as a cost-effective and scalable alternative for bathymetric mapping. By leveraging machine learning, the model mitigates the limitations of traditional empirical and physics-based approaches, offering improved accuracy in complex underwater terrains. This study underscores the feasibility of data-driven remote sensing for bathymetry estimation and provides a foundation for further advancements in machine learning with bathymetry applications.

## Figures and Tables

**Table 1.** Band and wavelength information for WV-2 multi-spectral images.

Bands	Wavelength (nm)
Coastal	400–450
Blue	450–510
Green	510–580
Yellow	585–625
Red	630–690
Red Edge	705–745
Near-IR1	770–895
Near-IR2	860–1040

**Table 2.** Comparison of 3-Fold, 5-Fold, and 10-Fold CV results by CatBoostOpt at Keaton Beach with raw reflectance values as inputs.

Cross-Validation	RMSE (m)	RMSE (%)	R^2^ (−)	MAE (m)	MSE (m^2^)	MAPE (%)
3-Fold	0.30	10.34	0.81	0.20	0.10	6.42
5-Fold	0.31	10.36	0.80	0.20	0.11	6.43
10-Fold	0.31	10.38	0.79	0.21	0.13	6.48

**Table 3.** Comparison of 3-Fold CV results (mean absolute error, MAE, in meters) on Sonar measurements at Saint Joseph Bay using different patch sizes.

Patch Size	RF (m)	Ada-Boost (m)	GB (m)	Linear SVM (m)
1×1×8	0.42	0.38	0.26	0.70
3×3×8	0.39	0.36	0.25	0.70
5×5×8	0.39	0.36	0.25	0.68

**Table 4.** CatBoostOpt model performance under different band configurations (8, 6, and 4 bands) at SJB, KB, and SGS locations.

# of Band	Metric (Unit)	SJB	KB	SGS	AVG
8 Bands	MAE (m)	0.25	0.19	0.25	0.23
	MSE (m^2^)	0.15	0.08	0.13	0.12
	RMSE (m)	0.39	0.32	0.37	0.36
	RMSE (%)	29.43	10.31	18.31	19.35
	R^2^ (−)	0.92	0.81	0.85	0.86
	MAPE (%)	33.80	6.31	12.72	17.61

6 Bands	MAE (m)	0.24	0.21	0.26	0.24
	MSE (m^2^)	0.15	0.11	0.15	0.14
	RMSE (m)	0.39	0.33	0.39	0.37
	RMSE (%)	28.98	10.92	18.44	19.45
	R^2^ (−)	0.91	0.78	0.83	0.84
	MAPE (%)	32.54	6.84	12.73	**17.37**

4 Bands	MAE (m)	0.27	0.29	0.29	0.28
	MSE (m^2^)	0.16	0.18	0.18	0.18
	RMSE (m)	0.40	0.43	0.43	0.42
	RMSE (%)	30.07	14.21	20.29	21.52
	R^2^ (−)	0.91	0.63	0.80	0.78
	MAPE (%)	46.36	9.45	14.69	23.50

**Table 5.** Three-fold CV results at SJB, KB, and SGS with raw reflectance values as inputs.

Location	Metric (Unit)	GB	RF	AdaBoost	SVM	LR	DCNN	CatBoost	CatBoostOpt
SJB	MAE (m)	0.28	0.26	0.38	0.36	0.66	0.68	0.25	0.25
	MSE (m^2^)	0.20	0.17	0.27	0.44	1.06	1.46	0.16	0.15
	RMSE (m)	0.45	0.41	0.52	0.66	1.03	1.21	0.40	0.39
	RMSE (%)	33.38	30.22	38.79	48.76	76.62	89.79	29.74	29.43
	R^2^ (−)	0.88	0.90	0.84	0.75	0.40	0.17	0.91	0.92
	MAPE (%)	41.09	33.87	97.61	43.81	105.33	154.60	34.13	33.80

KB	MAE (m)	0.22	0.20	0.28	0.22	0.32	0.22	0.21	0.19
	MSE (m^2^)	0.12	0.10	0.16	0.12	0.19	0.11	0.10	0.08
	RMSE (m)	0.35	0.32	0.40	0.35	0.44	0.33	0.32	0.32
	RMSE (%)	11.53	10.65	13.26	11.48	14.59	10.92	10.67	10.31
	R^2^ (−)	0.75	0.79	0.68	0.76	0.61	0.78	0.79	0.81
	MAPE (%)	7.16	6.56	9.36	6.91	10.64	6.92	6.67	6.31

SGS	MAE (m)	0.28	0.26	0.45	0.34	0.59	0.29	0.26	0.25
	MSE (m^2^)	0.18	0.16	0.33	0.24	0.61	0.17	0.15	0.13
	RMSE (m)	0.42	0.40	0.57	0.49	0.78	0.42	0.39	0.37
	RMSE (%)	20.00	18.93	27.12	23.36	37.20	19.86	18.61	18.31
	R^2^ (−)	0.80	0.82	0.63	0.73	0.31	0.80	0.83	0.85
	MAPE (%)	13.82	12.97	25.61	16.24	32.59	15.11	12.88	12.72

**Table 6.** Three-fold CV results at SJB, KB, and SGS with log-linear transformed raw reflectance values as inputs.

Location	Metric (Unit)	GB	RF	AdaBoost	SVM	LR	DCNN	CatBoost	CatBoostOpt
SJB	MAE (m)	0.29	0.24	0.38	0.34	0.64	0.66	0.24	0.23
	MSE (m^2^)	0.21	0.15	0.28	0.38	0.99	1.43	0.15	0.14
	RMSE (m)	0.46	0.38	0.53	0.61	0.99	1.20	0.38	0.36
	RMSE (%)	33.95	28.25	39.36	45.47	73.70	88.87	28.54	28.07
	R^2^ (−)	0.88	0.92	0.84	0.79	0.44	0.19	0.91	0.93
	MAPE (%)	41.58	35.27	97.17	45.34	100.61	153.77	33.53	33.26

KB	MAE (m)	0.22	0.20	0.28	0.22	0.32	0.23	0.21	0.18
	MSE (m^2^)	0.12	0.11	0.16	0.12	0.19	0.11	0.10	0.09
	RMSE (m)	0.34	0.32	0.40	0.35	0.44	0.33	0.32	0.30
	RMSE (%)	11.30	10.55	13.14	11.50	14.54	10.82	10.67	10.38
	R^2^ (−)	0.76	0.79	0.68	0.76	0.61	0.78	0.79	0.82
	MAPE (%)	7.13	6.81	9.31	6.93	10.59	6.84	6.69	6.46

SGS	MAE (m)	0.42	0.40	0.57	0.48	0.75	0.42	0.39	0.39
	MSE (m^2^)	0.17	0.16	0.33	0.23	0.56	0.17	0.15	0.15
	RMSE (m)	0.41	0.39	0.57	0.48	0.75	0.42	0.39	0.36
	RMSE (%)	19.70	18.88	27.19	22.86	35.62	19.80	18.60	18.28
	R^2^ (−)	0.81	0.82	0.63	0.74	0.37	0.80	0.83	0.85
	MAPE (%)	13.70	12.92	25.69	15.83	31.21	14.89	12.82	12.74

**Table 7.** Three-fold CV results at SJB, KB, and SGS with log-ratio transformed raw reflectance values as inputs.

Location	Metric (Unit)	GB	RF	AdaBoost	SVM	LR	DCNN	CatBoost	CatBoostOpt
SJB	MAE (m)	0.27	0.24	0.33	0.29	0.40	0.54	0.23	0.22
	MSE (m^2^)	0.20	0.15	0.20	0.22	0.36	0.85	0.14	0.13
	RMSE (m)	0.44	0.39	0.45	0.46	0.60	0.92	0.37	0.35
	RMSE (%)	32.90	28.38	33.51	34.45	44.79	68.72	27.56	27.34
	R^2^ (−)	0.89	0.92	0.88	0.88	0.79	0.51	0.92	0.94
	MAPE (%)	38.43	35.71	80.82	39.34	58.56	115.69	32.02	33.22

KB	MAE (m)	0.23	0.20	0.27	0.22	0.26	0.23	0.21	0.18
	MSE (m^2^)	0.12	0.10	0.15	0.12	0.30	0.12	0.10	0.09
	RMSE (m)	0.35	0.32	0.39	0.35	0.52	0.34	0.32	0.31
	RMSE (%)	11.53	10.60	12.75	11.61	17.03	11.36	10.62	10.48
	R^2^ (−)	0.75	0.79	0.70	0.75	0.40	0.76	0.79	0.82
	MAPE (%)	7.23	6.66	8.81	6.96	8.27	7.45	6.63	6.48

SGS	MAE (m)	0.28	0.25	0.37	0.30	0.40	0.30	0.26	0.24
	MSE (m^2^)	0.18	0.15	0.25	0.20	0.34	0.19	0.15	0.14
	RMSE (m)	0.43	0.39	0.50	0.45	0.58	0.43	0.39	0.37
	RMSE (%)	20.33	18.64	23.61	21.22	27.59	20.52	18.66	18.23
	R^2^ (−)	0.79	0.83	0.72	0.78	0.62	0.79	0.83	0.86
	MAPE (%)	13.93	12.56	19.99	14.22	20.87	15.43	12.63	12.43

**Table 8.** RMSE (m) between NOAA bathymetry and CatBoostOpt predictions at SJB, KB, and SGS.

Location	RMSE (m) Between NOAA and Model Predicted Bathymetry	
	Without Correction	With Seagrass Correction
SJB	0.50	0.56
KB	0.63	0.52
SGS	0.87	0.90

## Data Availability

In situ bathymetry and model data are available upon request from the authors. Satellite imagery can be obtained from the NASA Commercial Data Buy program or directly from Maxar. Remove the following statement from the Acknowledgements: The data will be made available by the authors on request.

## Abbreviations

The following abbreviations are used in this manuscript:

CV: Cross validation
DCNN: Deep convolutional neural network
GB: Gradient boosting
KB: Keaton Beach
LIDAR: LIght Detection Furthermore, Ranging
LR: Linear regression
MAE: Mean absolute error
lin NOAA: National Oceanic and Atmospheric Administration
RBF: Radial basis function
RF: Random forest
RMSE: Root mean square error
SAR: Synthetic aperture radar
SGS: Saint George Sound
SJB: Saint Joseph Bay
Sonar: SOund NAvigation Ranging
SVM: Support vector machine
WV-2: WorldView-2
